# Low serum vitamin-D status is associated with high prevalence and early onset of type-1 diabetes mellitus in Kuwaiti children

**DOI:** 10.1186/s12887-016-0629-3

**Published:** 2016-07-16

**Authors:** Majedah A. Rasoul, Maria Al-Mahdi, Hessa Al-Kandari, Gursev S. Dhaunsi, Mohammad Z. Haider

**Affiliations:** Department of Pediatrics, Faculty of Medicine, Kuwait University, PO Box 24923, Safat, 13110 Kuwait; Department of Pediatrics, Mubarak Al-Kabeer Hospital, Jabriya, Kuwait; Department of Pediatrics, Adan Hospital, Al-Adan, Kuwait; Department of Pediatrics, Farwania Hospital, Farwania, Kuwait

**Keywords:** Type-1 diabetes, Vitamin D, Kuwait, Deficiency

## Abstract

**Background:**

Type 1 diabetes mellitus (T1DM) is highly prevalent in Kuwait with incidence of around 40.1/100,000 individuals. Evidence indicate that vitamin D plays an important role in modulating the immune system and could thus impact the onset and high prevalence of T1DM. We report serum vitamin D levels in Kuwaiti children with T1DM and non-diabetic controls to explore its relationship with prevalence and onset of the disease.

**Methods:**

This study included 216 Kuwaiti Arab children with T1DM. The diagnosis of T1DM was based on the ISPAD criteria. The control subjects (204 Kuwaitis) were age and gender matched, healthy, non-diabetic, and had no close relative with T1DM. Vitamin D levels were determined in serum using an enzyme immunoassay (EIA) method.

**Results:**

The age of onset of T1DM was <4y in 20 % of the T1DM cases, between 4 and 6y in 28 % cases and >6y in 52 % patients. In T1DM patient group, 84 % subjects were found to be deficient in serum vitamin D level compared to 77 % of the controls (*p* = 0.046). Collectively, the deficient and insufficient vitamin D status was detected in 99 % of the T1DM patients compared to 92 % of the controls (*p* = 0.027). The mean serum vitamin D levels were found to be significantly different in early onset cases (age <4y) compared to the late onset sub-group (*p* = 0.001). A significant correlation was found between some elements of socioeconomic status, SES (i.e. parent’s profession and family’s income) and lower vitamin D levels in Kuwaiti T1DM children. There was no significant difference between mean serum vitamin D levels during winter and summer months in the T1DM patients.

**Conclusions:**

The proportion of cases with a deficient vitamin D status was significantly high in Kuwaiti T1DM children compared to the controls. The serum vitamin D levels were found to be significantly different in early onset and late onset T1DM patients. Therefore, serum vitamin D status can be considered an important contributor in high prevalence of T1DM in Kuwaiti children.

## Background

The incidence of type-1 Diabetes Mellitus (T1DM) has been increasing in Kuwait over the past three decades [1]. Shaltout et al. [2] reported a four-fold increase in the incidence of T1DM (15.4/100,000) in the 0–14y age-group compared to 3.96/100,000 individuals reported from Kuwait in 1980s [3]. In the period between 1992 and 93, the incidence was found to be 12.8/100,000 in 0–5y age group; 15.1/100,000 in 5–9y group and 18.3/100,000 in the 10–14y age group respectively [2]. Later in 2002, Shaltout et al. [1] reported an incidence of 20.1/100,000 Kuwaiti children in the age range 0–14 y. These authors also reported that in the 1980s, the incidence of T1DM in Kuwait was in the same range as ‘low incidence’ countries (3.96/100,000), however, during the subsequent 10 years period, in the 1992–93, it increased almost four times to 15.4/100,000 children [3] and was similar to that reported from ‘high incidence’ European countries [4, 5]. Patterson et al. [6] have documented the worldwide estimates of type 1 diabetes and reported an incidence of 22.3/100,000 in Kuwait. The most recent incidence data of T1DM in Kuwait comes from the National Type 1 Diabetes Registry (established in 2011); according to this source the incidence of T1DM in Kuwait is 40.1 per 100,000 individuals (Shaltout AA, Rasoul MA, Al-Khawari M; unpublished data). Rasoul et al. [7] also reported a very high incidence 20.1/100,000 during the period between 1995 and 99 in a hospital based study of the Kuwaiti T1DM children from a high population density area. Unlike other complex/chronic diseases of the childhood, a wide variation in the incidence of T1DM has been found in different world populations. The highest incidence has been reported from Finland and Sardinia [8] and low incidence was noted in Mexico, China, Korea and Japan [4]. The basis for such a wide variation in the incidence of T1DM in different populations and in the same population over time has been an area of intensive investigation. It has been suggested that environmental factors, nutritional pattern and life style changes have played a significant role in genetically susceptible population/ethnic groups which has resulted in rapid increase in the incidence of T1DM.

T1DM is considered to result from a complex interplay between predisposing genes, immune system mediators and environmental factors. An immune-mediated destruction of insulin-producing beta-cells in the pancreatic Islets of Langerhans is thought to result in T1DM. The activation of auto-reactive lymphocytes and the cytokine-induced apoptosis of pancreatic beta-cells play a major role in the etiology of T1DM. A variety of evidence indicate that vitamin-D plays an important role in modulating the immune system and could thus impact the onset of T1DM. Vitamin D is a potent immune-modulator, regulating cell proliferation and differentiation, lymphocyte activation and cytokine production [9]. 1,25-Dihydroxyvitamin-D_3_ [1,25(OH)_2_D_3_] inhibits lymphocyte activation and affects other elements of the immune system, such as cytokine and immunoglobulin production, as well as major histocompatibility (MHC) class-II and cluster of differentiation-4 (CD4) expression [10]. Recent evidence indicate that the production and degradation of 1,25(OH)_2_ vitamin-D is a major signaling component in both innate [11] and adaptive [12] immune systems. However, the relationship between circulating levels of 25(OH)-vitamin D and immune responsiveness is largely undefined [10]. In NOD mice, it has been shown that the development of diabetes can be prevented by administration of vitamin D [13]. Studies in humans have indicated that vitamin D supplementation in early childhood decreases the risk of T1DM and that intake of vitamin D in pregnancy may prevent the appearance of islet autoantibodies in the offspring [14–16]. The effect of vitamin D in T1DM was first proposed based on the observation that its incidence rates were negatively correlated with sunlight exposure, resulting in higher incidence at higher altitude [17], and the distinctive seasonal pattern in T1DM incidence with largest proportion of cases diagnosed in winter and lowest during summer [18]. In this prospective study, we report serum vitamin-D levels in Kuwaiti children with T1DM and healthy controls to explore its relationship with the disease.

## Methods

A total of 216 newly diagnosed T1DM patients were recruited from three major hospitals (Mubarak Al-Kabeer, Adan and Farwania). The inclusion criteria used was according to ISPAD/WHO protocol: i) diagnosis by a physician as diabetic; ii) placed on a daily dose of insulin before the 15th birthday and a Kuwaiti national resident in the area at the time of first insulin administration. In addition to the 216 T1DM patients a total of 204 non-diabetic controls were also studied. The selection of controls was random, they were all Kuwaiti Nationals and were matched for age, gender and ethnicity. The control subjects were healthy volunteers and a trained Diabetes specialist made a thorough assessment of their health status. This study has been approved by the Joint Committee for Protection of Human Subjects in Research of the Faculty of Medicine, Kuwait University and Kuwait Institute of Medical Specializations (KIMS), Ministry of Health, Kuwait. Informed consent was obtained from the study subjects and/or their parents as per regulations of the Ethics Committees.

### Collection and processing of samples

Approximately 5 ml blood was collected from all the study subjects in appropriate tubes for subsequent laboratory analyses. The blood was drawn from patients when they were first diagnosed with T1DM. The serum was isolated from samples collected in plain tubes and stored at −30 °C for subsequent analyses.

### Analysis of the biochemical parameters

Serum glucose levels were measured by routine work-up of T1DM patients by using glucose oxidase method, hemoglobin A1c (HbA1c) levels were measured by high performance liquid chromatography (HPLC), serum Ca^2+^ and phosphate concentration were determined using spectrophotometric methods.

### Measurement of 25-Hydroxyvitamin-D (25-OH Vitamin-D)

The levels of 25-Hydroxyvitamin-D in the serum were determined in T1DM patients and non-diabetic controls (216 patients and 204 controls respectively) by using a non-radioactive Enzyme Immunoassay kit (EIA kit, Immuno-Diagnostic Systems Ltd. Boldon, UK, www.idsplc.com). This method uses ‘Direct’ assay technology, eliminates the solvent precipitation and centrifugation steps thus resulted in automation of the ELISA procedure. It has been shown to possess excellent sensitivity (5 nmol/L, 2 ng/mL) from a small sample size (25 μl) and a wide assay range (6–360 nmol/L, 2.4–144 ng/mL). Furthermore, this method eliminated the need for using a radioactive tracer. Appropriate controls were included in all assays for standardization and quality control. Random samples were re-assayed in a quality control setting at Hospital laboratory to check the efficacy of the assay kits.

The vitamin-D levels in serum were considered *deficient* when <21 ng/ml; *insufficient* when 21–29 ng/ml and *sufficient* when >29 ng/ml as per guidelines of the Task Force of the Endocrine Society, 2011 [19]. The vitamin-D levels were determined in T1DM patients recruited in different months of the year and data was pooled from summer months (April-September) and winter months (October-March) for making comparisons.

### Detection of autoantibodies

Three autoantibodies, Islet Cell autoantibody (ICA), Insulin (INS) and glutamic acid decarboxylase (GAD) were detected in the serum samples from T1DM patients at the hospital laboratories by radioimmunoassay using commercial kits (Cisbio Assays, Codolet, France; www.cisbio.com).

### Documentation of socioeconomic status of the families with T1DM children

In order to obtain an assessment of the socioeconomic status (SES) of the Kuwaiti families with T1DM children, information was collected using a standardized questionnaire at the time of their recruitment in the study according to previously published strategy [20, 21]. The socioeconomic parameters/indicators included educational background of the parents, their profession and total monthly income of the family [20].

### Statistical analysis

The data was analyzed using the Statistical Package for the Social Sciences version 23 (SPSS, Chicago IL, USA). The serum vitamin D levels in different groups of subjects were presented as mean ± SD and comparisons between study groups were made by using Fisher’s Exact test, chi-square or student *t*-test. The *p*-values were considered significant when <0.05. In appropriate cases, odds ratios were calculated at 95 % confidence interval.

## Results

The characteristics of T1DM patients and controls are presented in Table 1. The Male/Female distribution was 104–112 in the T1DM patients group while it was 106–98 in the controls (the difference not statistically significant; *p* = 0.49; Table 1). The mean age of subjects in the T1DM patients group was 115.05 ± 38.44 months and that of the control group was 115.18 ± 32.7 months respectively (the difference was not statistically significant). The age of onset of T1DM was below 4y in 42/215 (20 %) patients; between 4 and 6y in 61/215 (28 %) patients and >6y in 112/215 (52 %) patients respectively (Table 1). Collectively, 48 % of the total number of T1DM patients included in this study can be considered to have an early onset of the disease while 20 % of the total number fall in the very early onset category (Table 1). In the T1DM patients, 32/179 (18 %) had an affected sibling; in 74/206 (36 %) cases, the parents were consanguineous while in 75/196 (38 %) cases even the grandparents were consanguineous (Table 1).

**Table 1:** Characteristics of Kuwaiti T1DM patients (*n* = 216) and controls (*n* = 204)

	T1DM patients	Controls	*p*-value	OR^a^	CI^b^
Gender					
Males	104	106	0.49	0.86	0.585–1.259
Females	112	98	0.49	1.165	0.794–1.709
Age at onset of T1DM (No. of subjects)					
< 4 y	42/215 (20 %)				
4–6 y	61/215 (28 %)				
> 6 y	112/215 (52 %)				
Family history in T1DM patients^c^					
Having an affected sibling	32/179 (18 %)				
Parents consanguineous	74/206 (36 %)				
Grandparents consanguinity	75/196 (38 %)				

^a^ *OR* odds ratio; ^b^ *CI* 95 % confidence interval; ^c^This information was not available for some of the cases

In 167/216 (77 %) T1DM patients, mean HbA1c was between 7 and 10 % (53–86 IFCC or mmol/mol) while 49/216 (23 %) T1DM patients had their HbA1c >10 %. All the control subjects had HbA1c in the normal range i.e. below 6 % (42 mmol/mol).

Insulin Cell auto-antibody (ICA) was detected in 124/209 (59 %) T1DM patients. INS autoantibody was found in 157/209 (75 %) of the patients while GAD was detected in 188/211 (89 %) T1DM cases respectively. In 87/209 (42 %) patients, all three autoantibodies were detected. In 6/209 (3 %) patients, no autoantibody was detected and of these patients who did not have any autoantibody, 5/209 (2 %) had HbA1c above 6.0 %.

The mean serum vitamin D level in Kuwaiti T1DM patients was 13.84 ± 6.66 ng/ml and in the controls it was 14.98 ± 10.4 ng/ml (*p* = 0.19). The status of serum 25-OH vitamin-D in T1DM patients and non-diabetic controls is presented in Table 2. In the T1DM patients group, 182/216 (84 %) were found to be ‘deficient’ in their vitamin-D status compared to 158/204 (77 %) of the controls (*p* = 0.046; OR 1.66; Table 2). The vitamin-D status was ‘insufficient’ in 31/216 (15 %) T1DM patients as compared to 29/204 (14 %) of the controls (*p* = 0.93). In only 3/216 (1 %) of the T1DM patients, the vitamin-D status was in the ‘sufficient’ range, compared to 11/204 (5 %) of the controls (*p* = 0.027; Table 2). When the ‘deficient’ and ‘insufficient’ sub-groups were taken together (213/216, 99 %) and compared to the controls (187/204, 92 %), the difference in the two study groups was found to be statistically significant (*p* = 0.027, OR, 4.176; Table 2).

**Table 2:** The vitamin-D status in Kuwaiti T1DM patients and controls (according to The Endocrine Society Guidelines; [19])

Sub-groups	T1DM patients (*N* = 216)	Controls (*N* = 204)	*p*-value	OR^a^	CI^b^
Deficient	182 (84 %)	158 (77 %)	0.046	1.66	1.02–2.701
Insufficient	31 (15 %)	29 (14 %)	0.93	0.977	0.565–1.689
Sufficient	3 (1 %)	11 (5 %)	0.027	0.239	0.0658–0.872
Defficient + Insufficient	213 (99 %)	187 (92 %)	0.027	4.176	1.147–15.202

^a^ *OR* odds ratio; ^b^ *CI* 95 % confidence interval

The mean serum vitamin D levels detected in the three sub-groups of T1DM patients on the basis of age of onset of the disease are presented in Table 3. The difference in the mean vitamin D level was found to be statistically significant between the early-onset sub-group (age of onset <4y) and the late onset sub-group (age of onset >6 y), *p* = 0.001 (95 % CI, 1.43 to 5.96).

**Table 3:** Serum vitamin D levels (ng/ml) in Kuwaiti T1DM patients divided into sub-groups on the basis of age of onset of the disease

Mean serum vitamin D levels ± SD* (ng/ml)				
<4 years (a) (*N* = 44)	2–6 years (b) (*N* = 55)	>6 years (c) (*N* = 115)	*p*-value	CI**
16.313 ± 6.877	14.56 ± 5.946	12.616 ± 6.566		
Statistical comparison between patient sub-groups				
Sub-group (a) with (b)			0.18	−8.30 to 4.336
Sub-groups (a) with (c)			0.001	1.43 to 5.96
Sub-group (b) with (c)			0.069	−0.15 to 4.03

^a^ *SD* standard deviation; ^b^ *CI* 95 % confidence interval

The socioeconomic status of the family was evaluated by obtaining information on the educational background of parents, their profession and the family’s total monthly income. Each of these indicators were then correlated with the mean vitamin D level in children with T1DM. The serum vitamin D level in Kuwaiti children with T1DM did not show any significant correlation with the educational background of the parents (Table 4). The data on correlation between mean serum vitamin D levels in T1DM children and parent’s profession and the family’s total monthly income is presented in Table 5. A statistically significant correlation was noted between the mean vitamin D level of T1DM children when the father’s profession was ‘semi-skilled’ and ‘Office workers’ (*p* = 0.05) and when their profession was ‘semi-skilled’ and ‘professional’ (*p* = 0.007; Table 5). In the case of mother’s profession, a statistically significant correlation was noticed in T1DM patient’s vitamin D levels when the mother’s profession was ‘laborer’ compared to ‘Office worker’ (*p* = 0.03); between ‘semi-skilled and ‘skilled’ (*p* = 0.002) and between ‘office worker’ and ‘skilled’ category (*p* = 0.045; Table 5). In the case of total monthly income, a statistically significant difference was found only between family’s income KD 1000 and income between KD 1000–2000 (*p* = 0.046). The differences between other income-groups were not statistically significant.

**Table 4:** Correlation of serum vitamin D level in Kuwaiti T1DM children with their parent’s education level

	Father’s education level			
Serum vitamin D levels in T1DM children	Illiterate (a) (*N* = 0)	Primary (b) (*N* = 26)	Secondary (c) (*N* = 78)	University (d) (*N* = 108)
^a^Mean serum Vitamin D levels (ng /ml)	–	13.729 ± 6.057	13.941 ± 6.33	13.965 ± 7.2
	Mother’s education level			
	(*N* = 3)	(*N* = 14)	(*N* = 50)	(*N* = 143)
^a^Mean serum Vitamin D levels (ng/ml)	8.23 + 6.471	12.898 + 5.224	14.763 + 8.373	13.888 + 6.071

^a^No statistically significant correlation was found between the mean serum vitamin D levels in Kuwaiti T1DM children and their parent’s education level

**Table 5:** Correlation of serum vitamin D level in Kuwaiti T1DM children with their parent’s profession and total family income

	Father’s profession					
	Not employed (a) (*N* = 22)	Laborer (b) (*N* = 6)	Semi-skilled (c) (*N* = 33)	Office worker (d) (*N* = 86)	Skilled (e) (*N* = 30)	Professional (f) (*N* = 33)
^a^Mean serum vitamin D levels (ng /ml)	14.590 ± 4.788	16.296 ± 4.876	11.305 ± 5.921	13.959 ± 7.238	13.512 ± 5.172	15.716 ± 7.658
	Mother’s profession					
	(*N* = 69)	(*N* = 4)	(*N* = 26)	(*N* = 21)	(*N* = 78)	(*N* = 12)
^b^Mean serum vitamin D levels (ng/ml)	13.561 ± 5.945	18.5 ± 4.2	10.877 ± 4.252	12.201 ± 5.392	15.43 ± 7.807	13.739 ± 6.385
	Family’s total income					
	< KD 1000, *N* = 4 (a)		KD 1000–2000, *N* = 97 (b)		> KD 2000, *N* = 110 (c)	
^c^Mean serum vitamin D levels (ng/ml)	7.795 ± 0.709		14.53 ± 0.619		13.539 ± 5.615	

^a^A statistically significant correlation was detected in serum vitamin D level between groups (c) and (d), *p* = 0.05) and between groups (c) and (f), *p* = 0.007 of the Father’s profession

^b^In case of ‘Mother’s profession, statistically significant correlation was found between groups (b) and (d), *p* = 0.03; between groups (c) and (e), *p* = 0.002 and between groups (d) and (e), *p* = 0.045)

^c^In the case of ‘Family’s total income, statistically significant correlation was noted only between groups (a) and (b), *p* = 0.046

The differences between all other groups based on parent’s profession and total family income were not statistically significant. The categories of the profession have been chosen as described previously by Shah et al. [20] in Kuwait for determining the socio-economic status (SES)

The seasonal distribution of mean serum vitamin-D levels in T1DM patients and controls is presented in Table 6. Serum vitamin D levels from T1DM patients and controls during different months of the year were analyzed after pooling the data from summer months (April-September) and winter months (October-March). The differences between the T1DM patients and controls were not statistically significant in these two sub-groups i.e. during the summer months (*p* = 0.228) and the winter months (*p* = 0.403).

**Table 6:** Serum vitamin D level (ng/ml; mean ± SD) detected in summer and winter months in Kuwaiti children with T1DM

Months (No. of subjects)	T1DM patients	Controls	*p*-value
^a^Summer months (73 patients, 61 controls)	14.51 ± 7.2	16.36 ± 9.77	0.228
^b^Winter months (143 Patients; 137 Controls)	13.49 ± 6.27	14.37 ± 10.65	0.403

^a^Summer months (April-September); ^b^Winter months (October-March)

## Discussion

The most striking finding in this study is that majority of the Kuwaiti T1DM children were found to be deficient in their serum vitamin-D status (84 %, Table 2). Collectively, the ‘deficient’ and ‘insufficient’ vitamin D status was detected in 99 % of the Kuwaiti children with T1DM (*p* = 0.027; Table 2). This is very high and somewhat surprising when considered together with the socioeconomic status (SES) of the families with T1DM children. In majority of the T1DM families, the parents were educated (Tables 4 and 5), had a high total monthly income and can be considered affluent. Therefore it appears that lack of awareness or proper attention to health matters by the parents, life style and family structure may be the contributing factors in poor vitamin-D status in these Kuwaiti T1DM children. This finding in our study that collectively the ‘deficient’ and ‘insufficient’ vitamin D status was found in 99 % Kuwaiti T1DM patients compared to 92 % in the controls (mentioned above) is in sharp contrast to a recent report from Saudi Arabia where the ‘deficient’ vitamin D status was found in 66.7 % Saudi T1DM patients compared to 41.7 % of the controls [22]. In another report from Saudi Arabia, vitamin D deficiency was detected in 84 % Saudi T1DM children compared to 59 % in the controls [23]. The prevalence of vitamin D deficiency in Kuwaiti T1DM patients and controls was much higher than that reported from similar studies in other World populations [24, 25]. Vitamin D deficiency was found to be 43 % in T1DM patients in a study from Australia [25], 60.5 % in Switzerland [26], 25 % in Italy [27] and 15 % in North America [24]. The report from Qatar [28] on the correlation of vitamin D deficiency with T1DM appear to be somewhat similar to our findings in Kuwait. In that study, vitamin D deficiency was reported in 90.6 % Qatari T1DM children compared to 85.3 % in the controls. In a Swedish study, serum 25 (OH) vitamin D levels were found to be similar in T1DM patients and controls in a cross-sectional study (sub-optimal in both groups; [29]). Another study from Sweden [30] reported low vitamin D levels in patients aged between 15 and 34 years who were newly diagnosed with T1DM compared to that in the controls. Similar findings have been reported from a study from India in newly diagnosed children and adolescents [31]. It is important to understand the impact of low vitamin D levels in T1DM patients because of its possible effect on the mechanism of autoimmune destruction of the β-cells. Therefore, determination of vitamin D status especially in newly diagnosed T1DM cases can be of benefit for prevention or delaying the insulin-dependence which can be achieved by supplementation with vitamin D or its analogues.

The effect of season on the vitamin-D status was evaluated by combining the data on vitamin-D levels in T1DM patients in summer and winter months. There appear to be no correlation between season and the mean vitamin-D levels in Kuwaiti T1DM patients and the controls. This is not surprising when one considers that due to harsh climate, the living in Kuwait is mostly indoors with little or no direct exposure to sunlight. It has been suggested in previous reports from the region [22, 29] that a strong relationship exists between vitamin D status and the clothing worn. Kuwait is an Arab country in which majority of both males and females wear traditional clothes which leaves very little areas of the body exposed. This coupled with the harsh desert climate (being confined to indoor living) is likely to result in poor vitamin D status in both the T1DM patients and non-diabetic controls.

Like other studies of this nature, our study has some limitations e.g. possibility of individual variability in nutritional habits and fat proportion in study subjects are difficult to ascertain. However, a stringent evaluation was carried out at the time of recruitment of the study subjects, which minimized the effects of these factors as much as possible.

Another important finding reported in this study is that in the T1DM patients group, 18 % children had another sibling who had the same disease. And in families having T1DM children, 36 % of the parent were consanguineous while in 38 % cases even the grandparents were also related. This phenomenon of inter-family marriages is common in many tribal societies (like in Kuwait) and should be considered an important factor that can significantly impact the ‘genetic factors’ contributing to onset of T1DM and together with other factors like vitamin D deficiency can explain its very high incidence (40.1/100,000) in Kuwait.

The vitamin-D deficiency can therefore be considered an important factor which has a significant role in high prevalence of T1DM in Kuwait. This highlights an urgent need for launching a campaign for both the care providers and general public to enhance awareness of this problem in order to reduce its negative impact on this highly prevalent disease which places considerable burden on health care system and the community.

## Conclusions

The deficient serum vitamin D status was found to be significantly high in Kuwaiti T1DM patients compared to that in the controls. Mean serum vitamin D levels were significantly different between the early onset T1DM patients and the late-onset cases. A significant correlation was detected between parent’s professions, family’s income and low vitamin D levels in Kuwaiti T1DM patients.

## Abbreviations

T1DM, type 1 diabetes mellitus; ISPAD, International Society for Pediatric and Adolescent diabetes; CD4, cluster of differentiation-4; HbA1c, hemoglobin A1c; HPLC, high performance liquid chromatography; KIMS, Kuwait Institute for Medical Specializations; EIA, enzyme immunoassay; ELISA, enzyme linked immune-sorbent assay; ICA, Islet cell autoantibodies; INS, insulin autoantibody; GAD, glutamic acid decarboxylase autoantibody; SES, socioeconomic status; SD, standard deviation; CI, confidence interval; KD, Kuwaiti dinar;
